# Comparison of Outcomes Between Transperitoneal and Retroperitoneal Robotic Partial Nephrectomy: A Meta-Analysis Based on Comparative Studies

**DOI:** 10.3389/fonc.2020.592193

**Published:** 2021-01-08

**Authors:** Daqing Zhu, Xue Shao, Gang Guo, Nandong Zhang, Taoping Shi, Yi Wang, Liangyou Gu

**Affiliations:** ^1^ Department of Urology, Hainan Hospital, Chinese PLA General Hospital, Sanya, China; ^2^ Department of Neurology, Hainan Hospital, Chinese PLA General Hospital, Sanya, China; ^3^ Department of Urology, Chinese PLA General Hospital, Beijing, China; ^4^ Department of Urology, Affiliated Hospital of Inner Mongolia University For Nationalities, Tongliao, China; ^5^ Department of Urology, The Second Affiliated Hospital of Hainan Medical College, Haikou, China

**Keywords:** kidney neoplasms, partial nephrectomy, robotics, outcome, meta-analysis

## Abstract

**Background:**

To compare perioperative, functional and oncological outcomes between transperitoneal robotic partial nephrectomy (TRPN) and retroperitoneal robotic partial nephrectomy (RRPN).

**Methods:**

A literature searching of Pubmed, Embase, Cochrane Library and Web of Science was performed in August, 2020. Pooled odds ratios (ORs) or weighted mean differences (WMDs) with 95% conﬁdence intervals (CIs) were estimated using ﬁxed-effect or random-effect model. Publication bias was evaluated with funnel plots. Only comparative studies with matched design or similar baseline characteristics were included.

**Results:**

Eleven studies embracing 2,984 patients were included. There was no signiﬁcant difference between the two groups regarding conversion to open (P = 0.44) or radical (P = 0.31) surgery, all complications (P = 0.06), major complications (P = 0.07), warm ischemia time (P = 0.73), positive surgical margin (P = 0.87), decline in eGFR (P = 0.42), CKD upstaging (P = 0.72), and total recurrence (P = 0.66). Patients undergoing TRPN had a significant higher minor complications (P = 0.04; OR: 1.39; 95% CI, 1.01–1.91), longer operative time (P < 0.001; WMD: 21.68; 95% CI, 11.61 to 31.76), more estimated blood loss (EBL, P = 0.002; WMD: 40.94; 95% CI, 14.87 to 67.01), longer length of hospital stay (LOS, P < 0.001; WMD: 0.86; 95% CI, 0.35 to 1.37). No obvious publication bias was identified.

**Conclusion:**

RRPN is more favorable than TRPN in terms of less minor complications, shorter operative time, less EBL, and shorter LOS. Methodological limitations of the included studies should be considered while interpreting these results.

## Introduction

Partial nephrectomy (PN) is suggested to be the standard management for renal tumor smaller than 4 cm. With the development of techniques, PN for large or complex renal tumors becomes more and more common. According to guidelines, PN is also recommended for T1b masses when technique feasible (1, 2). Due to the superior perioperative results and non-inferior oncological outcomes, evolution has progressed from open PN to minimally invasive partial nephrectomy including laparoscopic PN (LPN) and robotic PN (RPN) (3). Nevertheless, compared to the OPN, the longer warm ischemia time (WIT) and difficulties in tumor excision and suturing remain obstacles to the adoption of LPN. Because of the obvious advantages in instruments, robotic surgical system can be considered as the enhanced laparoscopy, which makes the challenging LPN procedures become easier and safer. Due to shorter length of hospital stay (LOS) and WIT, lower rate of conversion to radical surgery, better functional reservation (4), RPN has been increasingly adopted over LPN (5).

Just like laparoscopy, most early RPNs were performed through the transperitoneal approach (6). The increased space through the abdominal approach allows the arm to be spaced sufficiently to reduce external conflicts. Moreover, since being more familiar with anatomic landmarks in the abdominal cavity, most surgeons were prone to choose transperitoneal RPN. However, accessing posterior or lateral renal masses transperitoneally can be more difficult and needs more time and skillful technique (6). Furthermore, the transperitoneal approach may enhance the risk of intestinal damage, especially in patients who had history of abdominal surgery, and may lead to more pneumoperitoneal-related pain or a greater risk of intestinal obstruction (7). Hence, in some cases, the retroperitoneal approach can be a good alternative approach to RPN.

For RPN, both transperitoneal and retroperitoneal approaches have been well described and standardized. Since its the advantages and disadvantages of both approaches are being carefully examined, a debate is under way to determine their role. Several comparative studies have compared these two surgical approaches, and have reported some inconsistent results. Three systematic reviews and meta-analyses have compared the perioperative outcomes between transperitoneal and retroperitoneal RPN (8–10). However, new studies with more rigorous design have published recently, and non-comparable baseline characteristics may affect the results. Hence, we performed an update systematic review and meta-analysis about this topic. The perioperative, functional, and oncological results have been compared, and we only included comparative studies with matched design or similar baseline characteristics.

## Materials and Methods

The protocol of our study was registered in PROSPERO (No. CRD42020159718).

### Literature Search

Relevant studies were obtained by searching Pubmed, Embase, Cochrane Library and Web of Science in August, 2020 with no restriction to language. We used the search terms integrated subject relevant terms (kidney cancer, renal cell carcinoma, kidney or renal neoplasm, renal tumor) and intervention terms (robotic or robot-assisted, partial nephrectomy, nephron sparing surgery or operation, transperitoneal, retroperitoneal). Screening references of related literatures were also performed to identify potential missing studies.

### Inclusion and Exclusion Criteria

The primary inclusion criteria were studies that compared perioperative, functional and oncological results between transperitoneal robotic partial nephrectomy (TRPN) and retroperitoneal robotic partial nephrectomy (RRPN). Additional items of inclusion criteria included: (1) all patients were diagnosed with localized renal tumor; (2) comparative studies with matched design or similar baseline characteristics; (3) clear description of the surgical technique as TRPN or RRPN; (4) assessment of at least one of the outcomes of interest. When two or more studies were reported by the same center and/or authors, the most recent and comprehensive report was included.

The following exclusion criteria were applied: (1) not met the inclusion criteria; (2) focusing on pediatric patients; (3) partial nephrectomy only for benign tumor or solitary kidney; (4) non-original literatures (*eg*, review articles, systematic reviews, meta-analyses, letters, commentaries, abstract, thesis).

### Data Extraction

This process was performed by two independent researchers; disagreements were resolved by discussion. A predesigned form was used; the variables included first author’ name, year of publication, study design and setting, number of patients, mean age, gender ratio, mean BMI, mean tumor size, mean RENAL score, clinical T stage, follow-up duration, comparability, and perioperative, functional and oncological outcomes of interest.

The Newcastle-Ottawa Scale (NOS) (11) and the Risk Of Bias In Non-randomized Studies of Interventions (ROBINS-I) (12) were used to evaluate the quality of included studies.

### Outcomes of Interest

The following outcomes were applied to compare TRPN and RRPN. Only the endpoints reported by two or more studies were analyzed.

1. safety outcomes: conversion to open or radical surgery, total complication rate, Clavien–Dindo grade 1–2 and 3–5 complication rates.
2. effectiveness outcomes: operative time, warm ischemia time (WIT), estimated blood loss (EBL), length of hospital stay (LOS), positive surgical margin (PSM) rate, decline in estimated glomerular ﬁltration rate (eGFR), chronic kidney disease (CKD) upstaging, overall recurrence rate.

### Statistical Analysis

Meta-analyses were conducted with Review Manager v.5.3 (Oxford, UK). The weighted mean difference (WMDs) and 95% confidence intervals (CIs) were used for comparison of continuous variables. For dichotomous variables, odds ratios (ORs) and 95% confidence intervals (CIs) were applied for comparison. For literatures reporting continuous variables as median and range or interquartile range, we calculated the means and standard deviations (SDs) with the previous method (13). Statistical heterogeneity was evaluated by the chi-squared test with significance set at P <0.1, and the *I^2^* value was used to estimate the quantity of heterogeneity. When signiﬁcant heterogeneity displayed (P < 0.1 or *I^2^ > *50%), a random-effect model was used for outcomes; otherwise, a fixed-effect model was applied. When ten studies were included for outcomes, publication bias was assessed with funnel plot.

## Results

### Literature Search and Description of Eligible Studies

**Figure 1:**
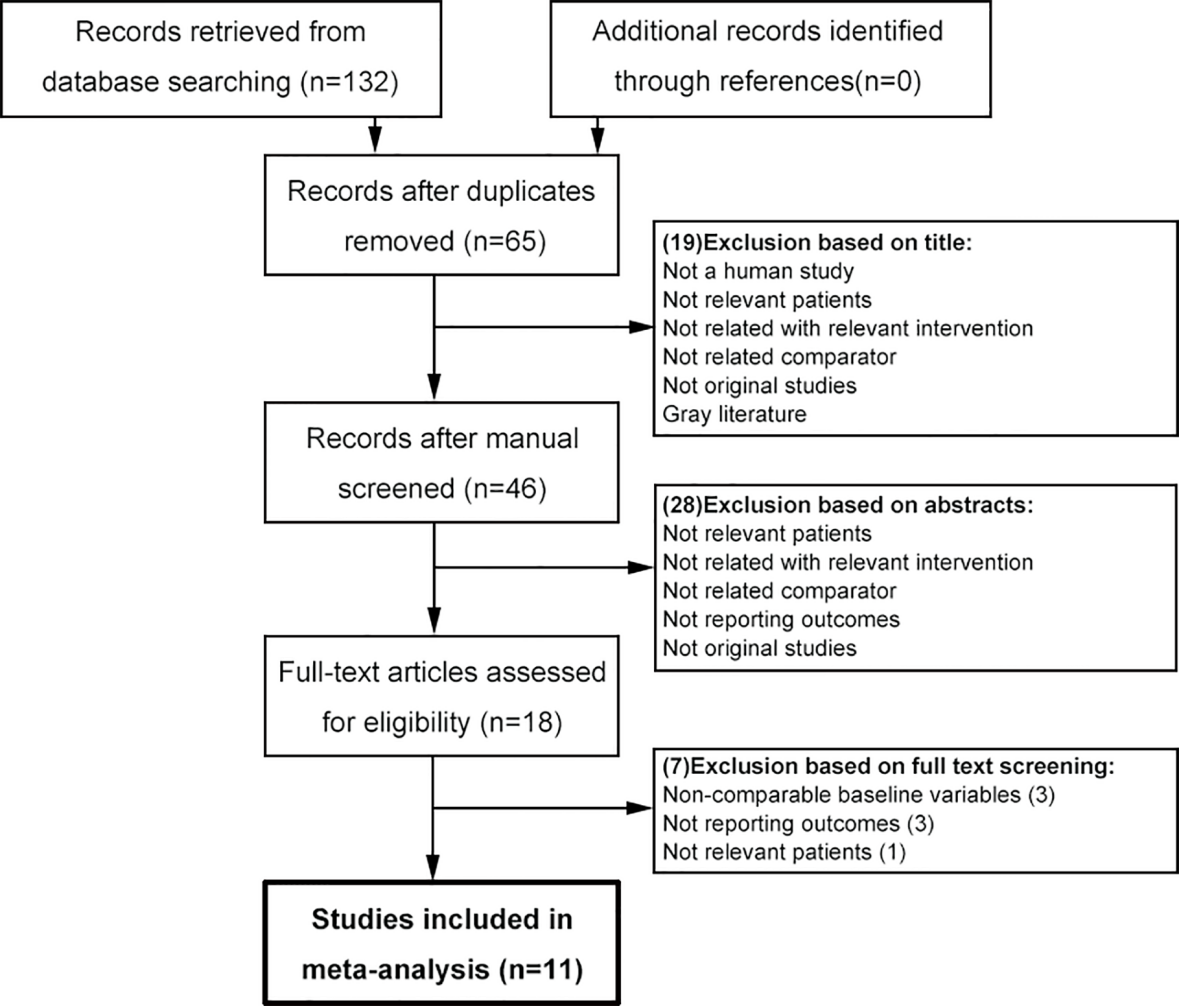
Flow-chart of literature searching.

The flow-chart of literature identification was shown in **Figure 1**. Database searching retrieved 132 studies, of which 67 were excluded due to duplicates, 19 and 28 were respectively excluded due to irrelevance based on title and abstract. Eighteen studies were assessed for eligibility with full-text, of which three were excluded due to non-comparable baseline characteristics, three were excluded due to no reporting outcomes, and one was excluded due to irrelevant patients. Lastly, 11 studies were included in the meta-analysis (6, 7, 14–22).

Demographics of included studies were described in **Table 1**. Three studies were prospective design, and eight studies were retrospective design, six studies included multi-institution subjects, and five studies included single-center subjects. Among the 1,715 patients undergoing TRPN, the mean age ranged from 49 to 62. Among the 1,269 patients undergoing RRPN, the mean age ranged from 51 to 61. All of them were comparative studies; six were performed with propensity score matching, five had similar baseline characteristics. The NOS scores for all included studies were seven or eight. All of the studies were classified as “moderate risk of bias” (**Table 2**). Detailed quality assessment conducted by Robins-I was shown in **Supplementary Table S1**. No significant differences were identified in regard to baseline variables between the two groups, including age, gender, BMI, tumor side and size, Charlson comorbidity index, ASA score, RENAL nephrometry score, tumor position, baseline eGFR, prior abdominal surgery (P > 0.05 for all) (**Table 3**).

**Table 1:** Demographics of included studies.

Study	Design	No. of patients		Mean age (years)		Gender (male/female)		Mean BMI (kg/m^2^)		Mean tumor size (cm)		Mean RENAL score	
		TP	RP	TP	RP	TP	RP	TP	RP	TP	RP	TP	RP
Takagi et al. (21)	Retro, SC	48	48	55	55	36/12	32/16	25	24	3.1	3	–	–
Paulucci et al. (14)	Pro, MI	157	157	60	61	97/60	103/54	29.4	29.2	3.1	3.1	7	7
Mittakanti et al. (6)	Retro, SC	166	166	60	60	–	–	30.3	29.7	3.3	3.1	6	6
Dell’ Oglio et al. (22)	Pro, MI	384	384	56	56	247/137	247/137	–	–	3	3	–	–
Choi et al. (15)	Retro, SC	60	31	49	51	35/25	22/9	25.5	25.3	4.8	4.6	7	7
Abaza et al. (16)	Pro, SC	107	30	56	54	56/51	18/12	32.4	30.7	3.5	3	7	7
Laviana et al. (17)	Retro, MI	78	78	57	59	53/25	51/27	–	–	–	–	7	7
Stroup et al. (18)	Retro, MI	263	141	58	59	158/105	82/59	28.6	29.8	3.1	2.9	7	7
Maurice et al. (19)	Retro, MI	296	74	59	58	168/128	51/23	29.2	29.7	2.6	2.5	7	7
Kim et al. (20)	Retro, SC	97	116	58	57	46/51	53/63	–	–	2.5	2.5	8	8
Hughes-Hallett et al. (7)	Retro, MI	59	44	62	61	–	–	–	–	3.6	3.2	6	6

TP, transperitoneal; RP, retroperitoneal; BMI, body mass index; Pro, prospective; MI, multi-institution; Retro, retrospective; SC, single center.

**Table 2:** Comparability and risk of bias for included studies.

Study	Clinical stage	Follow up duration, months		Comparability*	NOS score	ROBINS-I
		TP	RP			
Takagi et al. (21)	–	–	–	1, 2, 3, 6, 7, 8, 9, 10	7	Moderate
Paulucci et al. (14)	T1	20.3 (9.6–23.8)	23.0 (6.3–40.3)	1, 2, 3, 4, 5, 7, 8, 9, 10	8	Moderate
Mittakanti et al. (6)	–	–	–	1, 3, 5, 7, 8, 10	8	Moderate
Dell’ Oglio et al. (22)	T1-T2	–	–	1, 2, 4, 5, 7, 8, 9, 10	8	Moderate
Choi et al. (15)	T1b-T2a	32.5 (24.4)	39.2 (21.7)	1, 2, 3, 4, 6, 7, 8, 9	7	Moderate
Abaza et al. (16)	–	–	–	1, 2, 3, 4, 6, 7, 8, 11	7	Moderate
Laviana et al. (17)	–	8.3	7.1	1, 2, 3, 4, 6, 8, 9, 10	8	Moderate
Stroup et al. (18)	T1-T2	–	–	1, 2, 3, 4, 7, 8, 10	7	Moderate
Maurice et al. (19)	–	7.1 (0.5–24.0)	10.5 (2.6–24.9)	1, 2, 3, 4, 6, 7, 8, 9, 10	8	Moderate
Kim et al. (20)	–	–	–	1, 2, 3, 4, 5, 7, 8, 9, 10, 11	7	Moderate
Hughes-Hallett et al. (7)	–	–	–	1, 4, 7, 8	7	Moderate

TP, transperitoneal; RP, retroperitoneal; NOS, Newcastle-Ottawa Scale; ROBINS-I, Risk of Bias In Non-randomized Studies of Interventions.

*Comparability variables: 1 = age, 2 = gender, 3 = BMI, 4 = tumor side, 5 = Charlson comorbidity index, 6 = ASA score, 7 = tumor size, 8 = RENAL score, 9 = tumor position, 10 = baseline eGFR, 11 = prior abdominal surgery.

**Table 3:** Patients and tumor characteristics (transperitoneal *versus* retroperitoneal approach).

Variable	No. of studies where data available	Mean Difference/Odds ratio/Risk ratio	95% CI	P value
Age, years	11	−0.03	−1.00, 0.94	0.95
Male gender, n	9	0.94	0.79, 1.11	0.45
BMI, kg/m^2^	7	0.11	−0.47, 0.69	0.70
Right-sided tumor, n	9	1.01	0.86, 1.18	0.94
Charlson comorbidity index	2	−0.04	−0.30, 0.22	0.77
Tumor size, cm	10	0.16	-0.05, 0.27	0.53
RENAL score	9	−0.07	−0.25, 0.11	0.42
Posterior/lateral location, n	7	0.96	0.67, 1.36	0.80
Baseline eGFR, mL/min/1.73m^2^	8	−0.68	−2.29, 0.93	0.41
Previous abdominal surgery, n	2	0.81	0.51, 1.28	0.36

BMI, body mass index; eGFR, estimated glomerular filtration rate; CI, confidence interval.

### Safety Outcomes

**Figure 2:**
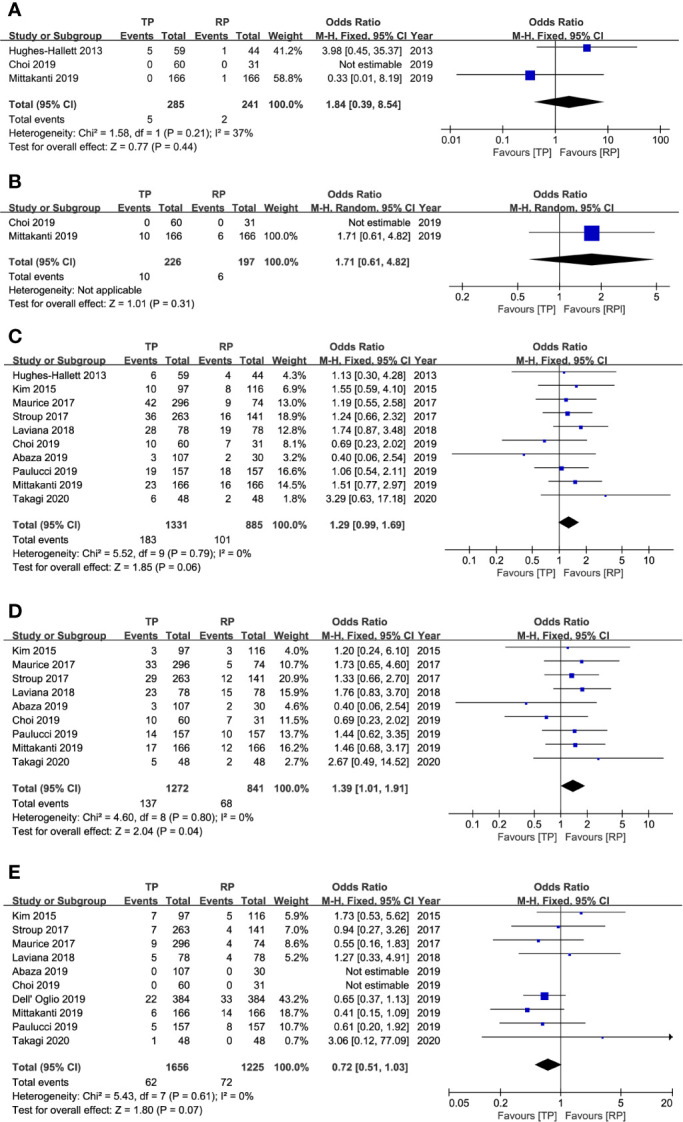
Forest plots of safety results. **(A)** conversion to open surgery; **(B)** conversion to radical surgery; **(C)** total complication; **(D)** Clavien–Dindo classification grades 1–2; **(E)** Clavien–Dindo classification grades 3–5.

The safety outcomes were presented in **Table 4** and **Figure 2**, including conversion to open or radical surgery, and surgical complications. There were no significant differences identified between the two groups in regard to conversion to open surgery (P = 0.44; OR: 1.84; 95% CI, 0.39–8.54), conversion to radical surgery (P = 0.31; OR: 1.71; 95% CI, 0.61–4.82), all Clavien–Dindo grade complications (P = 0.06; OR: 1.29; 95% CI, 0.99–1.69), Clavien–Dindo grade 3–5 complications (P = 0.07; OR: 0.72; 95% CI, 0.51–1.03). Patients undergoing RRPN had a lower rate of Clavien–Dindo grade 1–2 complications (P = 0.04; OR: 1.39; 95% CI, 1.01–1.91). No obvious publication bias was identified by funnel plots for total and major complications (**Figures 4A, B**).

**Table 4:** Safety results of TRPN and RRPN.

Study	No. of patients		Conversion to open (n)		Conversion to radical (n)		Total complication (n)		Complication, CD 1-2 (n)		Complication, CD 3-5 (n)	
	TP	RP	TP	RP	TP	RP	TP	RP	TP	RP	TP	RP
Takagi et al. (21)	48	48	–	–	–	–	6	2	5	2	1	0
Paulucci et al. (14)	157	157	–	–	–	–	19	18	14	10	5	8
Mittakanti et al. (6)	166	166	0	1	10	6	23	26	17	12	6	14
Dell’ Oglio et al. (22)	384	384	–	–	–	–	–	–	–	–	22	33
Choi et al. (15)	60	31	0	0	0	0	10	7	10	7	0	0
Abaza et al. (16)	107	30	–	–	–	–	3	2	3	2	0	0
Laviana et al. (17)	78	78	–	–	–	–	28	19	23	15	5	4
Stroup et al. (18)	263	141	–	–	–	–	36	16	29	12	7	4
Maurice et al. (19)	296	74	–	–	–	–	42	9	33	5	9	4
Kim et al. (20)	97	116	–	–	–	–	10	8	3	3	7	5
Hughes-Hallett et al. (7)	59	44	5	1	–	–	6	4	–	–	–	–

TRPN, transperitoneal robotic partial nephrectomy; RRPN, retroperitoneal robotic partial nephrectomy; TP, transperitoneal; RP, retroperitoneal; CD, Clavien–Dindo.

### Effectiveness Outcomes

**Figure 3:**
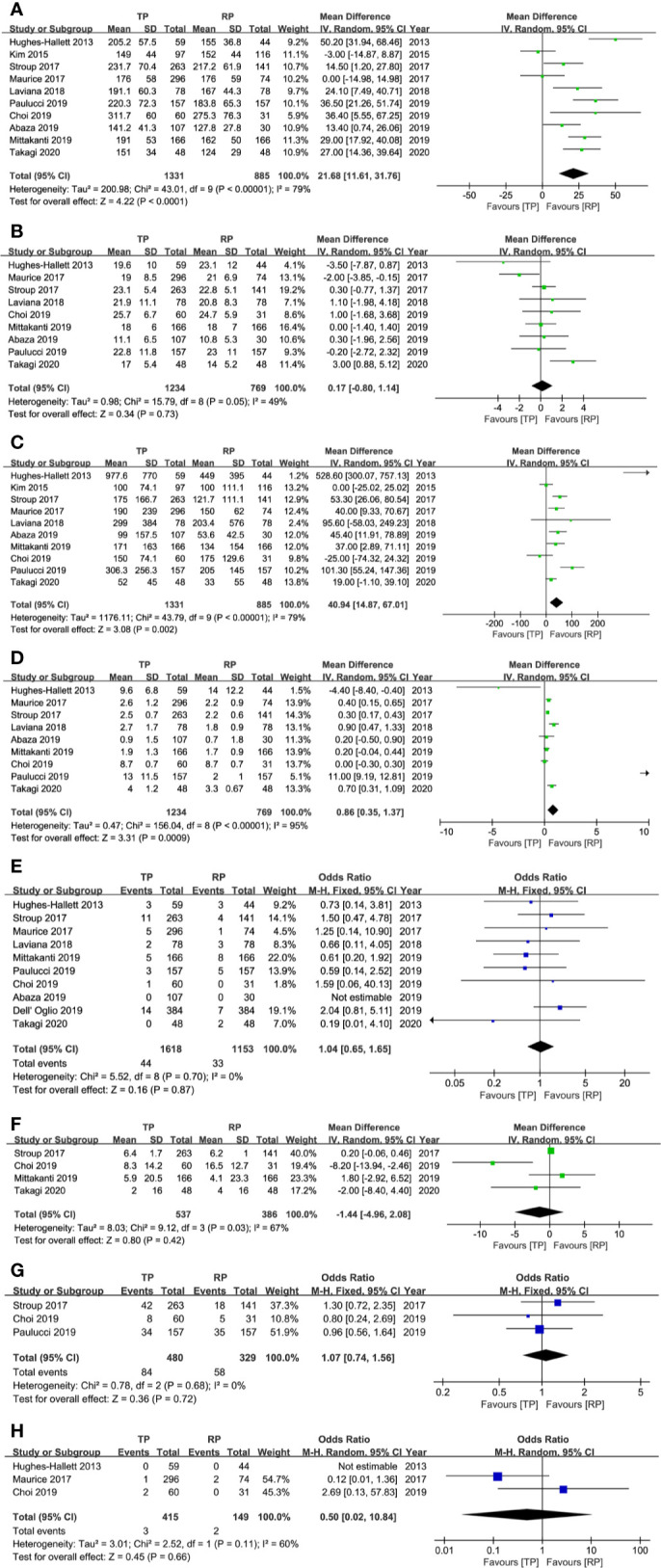
Forest plots of effectiveness results. **(A)** operative time; **(B)** warm ischemia time; **(C)** estimated blood loss; **(D)** length of hospital stay; **(E)** positive surgical margin; **(F)** decline of estimated glomerular ﬁltration rate; **(G)** chronic kidney disease upstaging; **(H)** overall tumor recurrence.

**Figure 4:**
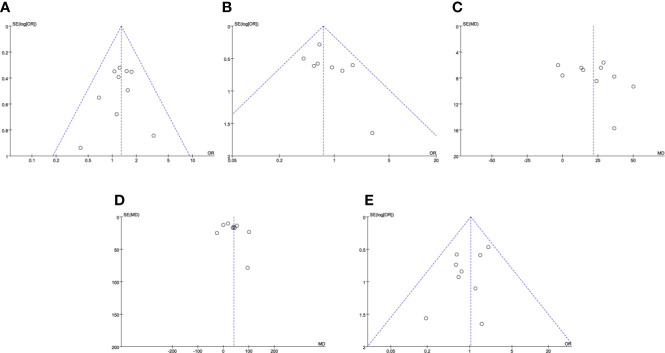
Funnel plots for results. **(A)** total complications; **(B)** Clavien–Dindo classification grades 3–5 complications; **(C)** operative time; **(D)** estimated blood loss; **(E)** positive surgical margin.

The effectiveness outcomes were presented in **Table 5** **and** **Figure 3**, including operative time, WIT, EBL, LOS, PSM rate, decline in eGFR, CKD upstaging, and total recurrence rate. There were no significant differences identified between TRPN and RRPN in terms of WIT (P = 0.73; WMD: 0.17; 95% CI, −0.80 to 1.14), PSM rate (P = 0.87; OR: 1.04; 95% CI, 0.65–1.65), decline in eGFR (P = 0.42; WMD: −1.44; 95% CI, −4.96 to 2.08), CKD upstaging (P = 0.72; OR: 1.07; 95% CI, 0.74–1.56), and total recurrence rate (P = 0.66; OR: 0.50; 95% CI, 0.02–10.84). Patients underwent TRPN had a longer operative time (P < 0.001; WMD: 21.68; 95% CI, 11.61 to 31.76), a more EBL (P = 0.002; WMD: 40.94; 95% CI, 14.87 to 67.01), a longer LOS (P < 0.001; WMD: 0.86; 95% CI, 0.35 to 1.37). There were moderate to high inter-study heterogeneity in operative time (*I^2^*= 79%), EBL (*I^2^*= 79%), LOS (*I^2^*= 95%), decline in eGFR (*I^2^*= 67%), recurrence rate (*I^2^*= 60%); however, subgroup analysis was not performed because of insufficient data. No obvious publication bias was identified with funnel plots for operative time, EBL, and PSM (**Figures 4C–E**).

**Table 5:** Effectiveness results of TRPN and RRPN.

Study	No. of patients		Mean OT (min)		Mean WIT (min)		Mean EBL (ml)		Mean LOS (d)		PSM (n)		eGFR, ml/min per 1.73 m^2^		CKD upstaging (n)		Recurrence (n)	
	TP	RP	TP	RP	TP	RP	TP	RP	TP	RP	TP	RP	TP	RP	TP	RP	TP	RP
Takagi et al. (21)	48	48	151	124	17	14	52	33	4	3.3	0	2	2	4	–	–	–	–
Paulucci et al. (14)	157	157	220.3	183.8	22.8	23	306.3	205	13	2	3	5	–	–	34	35	–	–
Mittakanti et al. (6)	166	166	191	162	18	18	171	134	1.9	1.7	5	8	5.9	4.1	–	–	–	–
Dell’ Oglio et al. (22)	384	384	–	–	–	–	–	–	–	–	14	7	–	–	–	–	–	–
Choi et al. (15)	60	31	311.7	275.3	25.7	24.7	150	175	8.7	8.7	1	0	8.3	16.5	8	5	2	0
Abaza et al. (16)	107	30	141.2	127.8	11.1	10.8	99	53.6	0.9	0.7	0	0	–	–	–	–	–	–
Laviana et al. (17)	78	78	191.1	167	21.9	20.8	299	203.4	2.7	1.8	2	3	–	–	–	–	–	–
Stroup et al. (18)	263	141	231.7	217.2	23.1	22.8	175	121.7	2.5	2.2	11	4	6.4	6.2	42	18	–	–
Maurice et al. (19)	296	74	176	176	19	21	190	150	2.6	2.2	5	1	–	–	–	–	1	2
Kim et al. (20)	97	116	149	152	–	–	100	100	–	–	–	–	–	–	–	–	–	–
Hughes-Hallett et al. (7)	59	44	205.2	155	19.6	23.1	977.6	449	4.6	2.5	3	3	–	–	–	–	0	0

TRPN, transperitoneal robotic partial nephrectomy; RRPN, retroperitoneal robotic partial nephrectomy; OT, operative time; WIT, warm ischemia time; EBL, estimated blood loss; LOS, length of hospital stay; PSM, positive surgical margin; eGFR, estimated glomerular ﬁltration rate; CKD, chronic kidney disease; TP, transperitoneal; RP, retroperitoneal.

## Discussion

Robotic partial nephrectomy can be conducted by transperitoneal or retroperitoneal approach. At first, due to technical difficulties of applying so many instruments in such small space of retroperitoneal cavity, TRPN was mainly performed over RRPN. For posterior or lateral renal masses, RPN through transperitoneal approach can be more difficult in assessing the tumor and suturing surgical wound (6). Several centers have reported these experiences about retroperitoneal RPN, and have confirmed the feasibility and safety of RRPN. Moreover, for selected patients, RRPN perhaps can achieve more favorable outcomes compared with TRPN, such as operative time, EBL, LOS (6, 16, 23). Recently, many newly published literatures also have studied this issue, and inconsistent results have been reported. In this case, a systematic review and meta-analysis were needed to overview these published literatures and provided more rigorous results.

Based on eleven comparative studies with matched design or similar baseline characteristics, the findings of the meta-analysis of 2,984 subjects presented that TRPN had a higher rate of minor complications, a longer operative time, a more EBL, a longer LOS compared to RRPN. The other results including conversion to open or radical surgery, rate of all or major complications, WIT, PSM rate, decline in eGFR, CKD upstaging, and total recurrence rate between the two approaches.

Previously, three meta-analyses have compared the perioperative outcomes between transperitoneal and retroperitoneal RPN. Xia et al. (9) initially have included four articles with a total of 449 patients to evaluate TRPN versus RRPN. There was no significant difference in any demographic variable between TRPN and RRPN, including tumor size and side, RENAL nephrometry score, and pathological type. They have found that only operative time was significantly different between TRPN and RRPN. The other outcomes were similar, including conversion, complication, WIT, EBL, PSM rate. Although comparable patients were included, four studies with only 449 patients was the main limitation. Then, Pavan et al. (8) have included seven retrospective studies with a total of 1,379 patients to compare perioperative outcomes between TRPN and RRPN. They have found that patients undergoing RRPN had a shorter operative time and LOS, a smaller EBL. The other outcomes were comparable, including postoperative complications, WIT, PSM rate. However, patients belonging to RRPN group had a larger tumor compared with TRPN. Incomparable baseline features, like tumor size may affect the surgical outcomes. More recently, McLean et al. (10) have performed a meta-analysis with only three literatures to compare these two approaches for posterior renal tumors, and only identified the advantage of RAPN in LOS.

Considering the comparability between the two groups, the present study only included comparative studies with matched design or similar baseline characteristics. Moreover, many studies about this topic have been published recently. The present study included six articles publishing in 2019 and 2020. Hence, based on rigorous and latest data, our study has identified the advantages of RRPN in minor complications, operative time, EBL and LOS. Furthermore, we have also compared functional and oncological outcomes between TRPN and RRPN. Limited data showed that decline in eGFR, CKD upstaging, and total recurrence rate were similar between the two approaches.

The significant difference in operative time was identified by the present study and previous two meta-analyses. The reduction in operative time for retroperitoneal approach may due to a more quick and direct access to kidney and hilum, which didn’t need to mobilize the adjacent bowel (7). Moreover, for posterior tumors, many surgeons also preferred to use a transperitoneal approach, which more time was required to access the posterolateral surface of the kidney and isolate tumors, especially in the presence of signiﬁcant adhesions and prior abdominal surgery. In this regard, a retroperitoneal approach can be a good alternative.

A lower EBL was another advantage for RRPN which was found in the present study. Nevertheless, though a significant difference was obtained in the analysis (P = 0.002), the clinical significance seemed to be limited (the difference was 41 ml). Hughes-Hallet et al. (7) have found a significantly higher median EBL in patients undergoing TRPN when compared with RRPN (395 ml *vs* 88 ml, P < 0.01). The authors explained that an early unclamping technique was more often used in the transperitoneal approach, and a less surgical dissection may be needed in the retroperitoneal approach. Abaza et al. (16) also reported a nearly two times mean EBL in patients undergoing TRPN when compared with RRPN (99 ml *vs* 54 ml, P < 0.001). The authors supposed that this may be caused by a less tissue dissection to access renal hilum, and a better identification and control of renal arteries or branches because of the approach. However, there was no study distinguished EBL during tumor resection and suture from EBL during tissue dissection, hence, it is difficult to make it clear (8, 24).

The present study also found that a nearly one-day shorter LOS in the RRPN group (P < 0.001; WMD: 0.86; 95% CI, 0.35 to 1.37). Using a multi-institutional RPN database, after propensity-score matching, Maurice et al. (19) have found a longer mean LOS in patients undergoing TRPN when compared with RRPN (2.6 *vs* 2.2 days, P = 0.01). A faster recovery of bowel function may partly explain this difference. Similarly, using a matched-pair design, Laviana et al. (17) reported a mean of 1.8 days in the RRPN group *versus* 2.7 days in the TRPN group (P < 0.001). Moreover, they found that patients undergoing TRPN were two and half times likely to experience a LOS longer than 2 days compared with RRPN (P = 0.014). A further cost analysis identified that a shorter operative time and LOS were the key factors which lead to a reduced cost in the RRPN group. More recently, Paulucci et al. (14) have described similar result; they supposed that the reduced LOS may be related to the shorter operative time, and a reported faster bowel function recovery. Additionally, the LOS may be affected by many other factors besides surgical approach, including age, gender, patient comorbidity, baseline renal function, tumors’ characteristics, and surgeons’ experience. Nevertheless, Kim et al. (20) have found that surgical approach was independent predictor for LOS longer than one day (OR: 7.4; P < 0.01) in the multivariate analysis. Finally, length of hospital stay is complex process affected by many components, and the surgical approach only stands for one factor in this process.

The limitations of the present study also needed to be addressed. The major one is all included studies were non-randomized designed. Although it represents a powerful statistical tool, meta-analysis is greatly affected by the included studies. Due to the non-randomized design, potential selection bias may have an influence on the surgical outcomes. Considering these, we preferred to include comparative studies with matched design. Due to limited studies, we also included studies with similar baseline characteristics. In this case, some variables such as posterior/anterior location may not be balanced in the two groups. Four included studies only analyzed patients with posterior tumors. Generally, most surgeons prone to choose transperitoneal approach for anterior tumors, and retroperitoneal approach for posterior tumors. Moreover, related data was limited, especially for some specific outcomes, including conversion, decline in eGFR, CKD upstaging, and recurrence rate. Lastly, some studies only provided median and IQR/range data for some variables, we estimated mean and SD using previous reported methods, which may be not so accurate. Despite these limitations, the present study stands for the latest, and most comprehensive and rigorous systematic review and meta-analysis on this topic.

## Conclusions

Our findings presented that RRPN is correlated with more pleasant outcomes than TRPN in regard to lower rate of minor complications, shorter operative time, less EBL, and shorter LOS. There was not a significant difference between TRPN and RRPN regarding recurrence rates, PSM and functional outcomes. Randomized studies with good design are needed to validate safety and effectiveness results of RRPN.

## Data Availability Statement

The original contributions presented in the study are included in the article/**Supplementary Material**, further inquiries can be directed to the corresponding authors.

## Author Contributions

DZ, YW, and LG conceptualized and designed the study. DZ, XS, and GG collected and managed the data. DZ, XS, and NZ analyzed the data. DZ, TS, YW and LG wrote and edited the manuscript. All authors contributed to the article and approved the submitted version.

## Conflict of Interest

The authors declare that the research was conducted in the absence of any commercial or financial relationships that could be construed as a potential conflict of interest.
