# Supplementary material for: Comparison of Outcomes Between Transperitoneal and Retroperitoneal Robotic Partial Nephrectomy: A Meta-Analysis Based on Comparative Studies
Source: Front Oncol. 2021 Jan 8;10:592193. doi: 10.3389/fonc.2020.592193 (PMC7819878; doi:10.3389/fonc.2020.592193)
Supplement: Supplementary file 1 [file Table_1.docx]

Table S1: Results of the assessment of risk of bias in included studies

| Study ID | 1. Bias caused by confounding | 2. Bias caused by  selection of  participants | 3. Bias caused by  classification of  interventions | 4. Bias caused by  deviations from  intended  interventions | 5. Attrition bias  caused by  missing data | 6. Detection bias  caused by  measurement of  outcomes | 7. Reporting bias  caused by  selection of the  reported results | Overall  judgement |
| --- | --- | --- | --- | --- | --- | --- | --- | --- |
| Takagi 2020 | MODERATE | MODERATE | MODERATE | LOW | MODERATE | MODERATE | LOW | MODERATE |
| Paulucci 2019 | LOW | MODERATE | MODERATE | LOW | LOW | MODERATE | LOW | MODERATE |
| Mittakanti 2019 | MODERATE | SERIOUS | MODERATE | LOW | LOW | MODERATE | LOW | MODERATE |
| Dell' Oglio 2019 | MODERATE | MODERATE | MODERATE | LOW | LOW | MODERATE | LOW | MODERATE |
| Choi 2019 | MODERATE | MODERATE | MODERATE | LOW | MODERATE | MODERATE | LOW | MODERATE |
| Abaza 2019 | MODERATE | SERIOUS | MODERATE | LOW | LOW | MODERATE | LOW | MODERATE |
| Laviana 2018 | MODERATE | MODERATE | MODERATE | LOW | LOW | MODERATE | LOW | MODERATE |
| Stroup 2017 | MODERATE | SERIOUS | MODERATE | LOW | LOW | MODERATE | LOW | MODERATE |
| Maurice 2017 | MODERATE | MODERATE | MODERATE | LOW | LOW | MODERATE | LOW | MODERATE |
| Kim 2015 | MODERATE | MODERATE | MODERATE | LOW | LOW | MODERATE | LOW | MODERATE |
| Hughes-Hallett 2013 | MODERATE | SERIOUS | MODERATE | LOW | MODERATE | MODERATE | LOW | MODERATE |
